# Recommendations for perioperative management of lung cancer patients with comorbidities

**DOI:** 10.1007/s11748-017-0864-z

**Published:** 2017-11-16

**Authors:** Hiroyoshi Tsubochi, Tomoki Shibano, Shunsuke Endo

**Affiliations:** Department of Thoracic Surgery, Jichi Medical University, Tochigi, Japan

**Keywords:** Lung cancer, Pulmonary resection, Idiopathic pulmonary fibrosis, Cardiovascular disorders, End-stage renal disease

## Abstract

**Objectives:**

To improve surgical outcomes, clinicians must provide optimal perioperative care for comorbidities identified as significant factors in risk models for patients undergoing lung cancer surgery.

**Methods:**

We reviewed trends in perioperative care for idiopathic pulmonary fibrosis, cardiovascular diseases, and end-stage renal diseases in patients undergoing lung cancer surgery, as large clinical databases indicate that these comorbidities are significant risk factors for lung cancer surgery. Articles identified by keyword searches were included in the analysis.

**Results:**

Significant predictive factors for acute exacerbation of idiopathic pulmonary fibrosis were identified. However, no effective perioperative care was identified for prevention of acute exacerbation of interstitial pneumonia. The timing of coronary revascularization and antithrombotic management for cardiovascular diseases are subjects of ongoing research, and acid–base balance is essential in the management of hemodialysis patients with end-stage renal diseases.

**Conclusions:**

To improve surgical outcomes for lung cancer patients, future studies should continue to study optimal perioperative management of comorbidities.

## Introduction

Lung cancer is a leading cause of death worldwide, and surgery remains a mainstay for complete cure. The main determinants of patient eligibility for surgical treatment are lung cancer stage, performance status, and parameters of pulmonary function. Recent studies of large clinical databases related to lung cancer surgery indicate that comorbidities affect operative mortality and morbidity. Risk model analysis showed that cardiovascular disorders, end-stage renal failure, and interstitial pneumonia are significant risk factors in operative mortality and morbidity [1]. Preoperative assessment of these risk factors might aid decisions regarding surgical treatment, and perioperative management of these comorbidities is necessary in order to improve surgical outcomes for lung cancer patients.

This review discusses findings from recent studies of trends in specialized perioperative care for comorbidities such as idiopathic pulmonary fibrosis (IPF), cardiovascular diseases (CVD), and end-stage renal diseases (ESRD) in patients undergoing lung cancer surgery.

## Search strategy

A search of large clinical databases related to lung cancer surgery risks identified the comorbidities of coronary disease, IPF, and end-stage renal failure as risk factors associated with mortality and morbidity after lung cancer surgery. This article reviews evidence from recently published papers included in PubMed on current management of IPF, CVD, and ESRD.

## Results

### IPF

IPF, a form of interstitial pneumonia (IP), is a progressive fibrotic lung disease of unknown cause. Incidence is higher in men, smokers, and persons with lung cancer. According to the Japan National Clinical Database (NCD), the IP incidence rate among patients who underwent pulmonary resection for lung cancer was 4.7% during 2014–2015 [1]. Some IP patients with lung cancer develop acute exacerbation (AE) after pulmonary resection, a major complication that can lead to death. Treatment of AE is difficult because of the rapid, aggressive progression to respiratory failure and the absence of definitive treatment and management options. A risk model for lung cancer surgery based on NCD data indicates that IP is an important risk comorbidity for operative mortality and morbidity [1]. Therefore, the presence of IP must be considered in surgical decision-making, and perioperative management is essential for improving clinical outcomes.

#### Clinical assessment

The pathological findings of IPF show usual IP (UIP) since American Thoracic Society and European Respiratory Society (ATS/ERS) proposed international clarification of IP [2]. The radiological features (subpleural, basal predominant honeycomb with septal thickening, traction bronchiectasis, and ground glass attenuation lesions) were clarified in relation to the pathological findings of IPF. More recently, features of definite IPF on high-resolution computed tomography (HRCT) images have a high specificity for the UIP pathological pattern, without surgical lung biopsy [2].

#### Acute exacerbation

Acute exacerbation of IPF (AE-IPF) is the rapid progressive deterioration of the lungs in patients with IPF. The clinical course is specific and distinguishable from those for infectious pneumonia, pulmonary emboli, and cardiogenic pulmonary edema [3]. AE-IPF often requires intensive care and is associated with high mortality. AE-IPF incidence was highly variable in previous studies. A cohort study of 594 IPF patients showed an AE incidence of 9.8% over a 10-year period [4]. AE-IPF sometimes occurs during pulmonary resection for patients with lung cancer associated with IPF, perhaps because of temporary pulmonary hypertension or exposure to hyperoxia, barotrauma, or volutrauma enhanced by mechanical ventilation. A large multicenter cohort study of 1763 lung cancer patients with interstitial lung disease who underwent pulmonary resection reported that 9.3% of patients developed AE and that the mortality rate was 43.9% [5]. Risk factors for AE-IPF included lower vital capacity, smoking, pulmonary hypertension, and more extensive lesions on HRCT. Surgical procedure, male sex, history of AE, preoperative steroid use, KL-6 level, a UIP pattern on computed tomography (CT), and reduced percent predicted vital capacity were identified as independent risk factors for AE.

#### Prophylaxis

Prophylactic management should be considered for lung cancer patients at high risk of AE-IPF after lung cancer surgery. Using data on preoperative patient characteristics and surgical procedure, the Japanese lung cancer scoring system yields a risk ratio for AE-IPF (Table 1) [6]. Positron-emission tomography (PET)/CT findings may be a marker of IPF severity and predict progression-free survival in patients with IPF [7].

**Table 1:** Risk scoring system for predicting acute exacerbation of interstitial pneumonia after pulmonary resection in lung cancer patients

History of acute exacerbation	5
CT findings: UIP pattern	4
Surgical procedure > wedge resection	4
Preoperative steroid use	3
Male gender	3
KL-6 > 1,000 U/mL	2
% vital capacity	1

Grade of risk	Risk score
Low risk < 10%	< 11
Intermediate risk < 10–25%	11–14
High risk < 25%	> 14

##### Pirfenidone

Several types of drugs (e.g., steroids, erythromycin, and neutrophil elastase inhibitors) have been proposed to prevent AE-IPF onset after surgery. However, no consensus exists regarding definitive prophylaxis for AE-IPF after pulmonary resection. Oral pirfenidone, a pyridone compound with pleiotropic effects, exhibited antioxidant, anti-inflammatory, and antifibrotic effects in experimental models of pulmonary fibrosis. Pirfenidone might lower levels of factors associated with proliferation, such as transforming growth factor-beta [8]. A phase II study found that pirfenidone limited IPF disease progression and lowered AE-IPF incidence after lung cancer surgery [9]. However, these findings require confirmation in a larger, prospective phase III study.

#### Preoperative evaluation

Prediction of postoperative complications and long-term disability caused by pulmonary resection requires preoperative physiologic evaluation, to determine surgical indications for lung cancer patients with underlying pulmonary diseases such as IPF or chronic obstructive disease. Spirometry is an essential component of the functional workup for surgical candidates. Among the characteristics to be evaluated, forced expiratory volume in 1 s (FEV_1_) and diffusing capacity for carbon monoxide (D_LCO_) are useful in predicting postoperative risk and long-term disability. Predicted postoperative (PPO) FEV_1_ and PPO D_LCO_ are associated with increased morbidity and mortality rates. D_LCO_ might help predict AE-IPF if it is evaluated. A functional algorithm that includes PPO FEV_1_ and PPO D_LCO_ was proposed previously [10]. When PPO FEV_1_ and PPO D_LCO_ are both greater than 60%, the risk of anatomic lung resection is low and no further tests are necessary. When PPO FEV_1_ or PPO D_LCO_ is less than 60% and greater than 30%, a stair-climbing or shuttle walk test should be performed. The risk of anatomic resection is low when stair-climbing altitude is greater than 22 m or shuttle walk distance is greater than 400 m. When PPO FEV_1_ or PPO D_LCO_ (or both) is less than 30% or the result of a stair-climbing or shuttle walk test is unsatisfactory, measurement of peak oxygen consumption (VO_2_ max) is recommended. A VO_2_ max less than 10 mL/kg/min or less than 35% predicted indicates a high risk of cardiopulmonary complications and mortality after major anatomical lung resection. Sublobar resection or a nonoperative modality should be considered for patients with a low VO_2_ max.

#### Operative care

Several factors may interact in the onset of AE-IPF after lung cancer surgery, including lung volume loss, mediastinal dissection, positive pressure ventilation, one-lung ventilation, high oxygen concentration exposure, overloaded fluid volume, blood loss, transfusion, and operation time.

##### Surgical approach

Even though a minimally invasive approach is recommended in order to hasten recovery from surgical stress, the benefit in reducing AE-IPF incidence is unclear [5]. One-lung ventilation is usually used for minimally invasive surgery, but could increase the risk of acute lung injury [11].

##### Fluid management

Excessive fluid infusion can cause pulmonary edema under normal hydrostatic pressure, a reported risk factor for acute lung injury after thoracic surgery. A previous study reported that 7–8 mL/kg/h is the proper intraoperative fluid infusion rate for patients undergoing pulmonary resection [12].

##### One-lung ventilation and oxygen concentration

A Japanese cohort survey found that the incidence rate of AE was 25.2% for the contralateral lung and 60.6% for both lungs among patients developing AE-IPF after lung cancer surgery [5]. These findings suggest that exposure to hyperoxia, barotrauma, and volutrauma associated with selective mechanical ventilation has a critical role in AE-IPF onset [12]. The synergy between lung-damaging noxious agents is mediated by oxygen radicals. Oxygenation may weaken the hypoxia-driven anti-inflammatory mechanism in local tissue, thereby exacerbating lung injury [13]. The minimum required oxygen concentration should be adjusted during surgery by using intermittent bilateral ventilation. High airway pressures or tidal volumes during one-lung ventilation can lead to barotrauma and volutrauma in the contralateral lung. Low-pressure controlled one-lung ventilation can relieve such stress [14]. These management decisions should be made in consultation with an anesthesiologist.

##### Extent of pulmonary resection

Pulmonary resection more extensive than partial resection is a risk factor for AE-IPF [5, 6]. However, it is unclear if the prognosis is better for limited resection than for standard resection. Underlying diseases associated with lung cancer, such as IPF, may be more advanced than clinical assessments indicate [15]. Thus, the extent of pulmonary resection should be carefully considered.

##### Nodal dissection

Nodal dissection increases tissue damage by reducing lymphatic drainage and bronchial circulation and denervating the vagal nerve, thus increasing the morbidity rate after lung cancer surgery [16]. The extent of nodal dissection was significantly related to AE-IPF incidence after lung cancer surgery [5]. Systematic nodal dissection should be indicated by preoperative PET assessment and nodal assessment by transbronchial biopsy through endobronchial ultrasonography. If mediastinal nodal involvement is diagnosed, surgical indications should be carefully reviewed. In particular, the prognoses of IPF and lung cancer with nodal involvement must be compared.

#### Postoperative care

##### Oxygen therapy

Oxygen therapy is often required for postoperative hypoxia. However, there is no evidence of benefit for oxygen treatment in patients with chronic hypoxia caused by IPF. Oxygen therapy should be limited unless it is necessary for recovery from limitations in postoperative activity.

##### Antibiotics

The role of respiratory infection in AE-IPF is not well understood. Empiric use of antibiotics is largely attributable to the fact that AE-IPF cannot always be distinguished from bacterial infection. Procalcitonin, a peptide more frequently observed in the setting of microbial toxins and bacterial proinflammatory molecules, is useful in detecting whether the cause of inflammation is bacterial in origin and in guiding the initiation and discontinuation of antibiotics in acute respiratory infections. Antibiotic therapy guided by procalcitonin at a threshold of 0.25 ng/mL reduced antibiotic treatment duration [17]. Azithromycin, an antibiotic with anti-inflammatory properties, was evaluated as an IPF treatment and significantly reduced fibrosis and restrictive lung function pattern in a mouse model of bleomycin-induced pulmonary fibrosis [18]. To date, no formal clinical trial has examined azithromycin for patients with AE-IPF.

##### Fluid infusion and blood transfusion

Liberal fluid treatment increases the incidence of acute lung injury. The volume of fluid administration should be adjusted at 1–2 mL/kg/h to maintain blood pressure and urine volume at > 0.5 mL/kg/h [19]. A positive daily fluid balance of 1.5 L should not be exceeded. Blood transfusion was reported to induce transfusion-related acute lung injury, which is distinguishable from AE-IPF but nevertheless a concern.

##### Planned follow-up CT

The need for chest CT is usually based on patient clinical conditions, such as dyspnea, decreased oxygen saturation (SpO_2_), abnormal shadows on chest radiography, and abnormal findings on blood tests. Scheduled chest CT examinations on postoperative days 4, 7, and 14 could hasten detection of AE-IPF [20]. Earlier treatment could decrease AE-IPF mortality. Scheduled chest CT should be considered for lung cancer patients at high risk, as determined by a scoring system for predicting AE-IPF after pulmonary resection.

##### Inhaled *N*-acetylcysteine monotherapy

*N*-Acetylcysteine (NAC) is a mucolytic antioxidant drug and has been studied as a potential treatment for IPF. It is hoped that NAC can prevent oxidative injury preceding fibroproliferation by restoring the natural oxidant/antioxidant balance. NAC inhalation monotherapy has not been studied as a prophylactic drug for AE-IPF, although favorable effects on lung function have been reported for patients with mild IPF, especially when NAC is combined with antifibrotic agents [21].

### CVD

As the population continues to age, an increasing number of patients with heart disease are undergoing non-cardiac surgery. Guidelines have been developed for perioperative management of patients undergoing non-cardiac surgery, among which the American College of Cardiology/American Heart Association (ACC/AHA) Guidelines on perioperative cardiovascular evaluation for non-cardiac surgery are most important [22]. The Scientific Committee of the Japanese Circulation Society has recently presented an original guideline that adopts the ACC/AHA Guidelines [23].

#### Ischemic heart disease

##### Timing of cardiac revascularization for patients with ischemic heart disease (IHD) who undergo non-cardiac thoracic surgery

The Revised Cardiac Risk Index is widely used to predict the risk of cardiovascular complications and death (Table 2). Patients with high cardiovascular risk require appropriate interventions before surgery. However, the benefit of coronary artery revascularization before elective non-cardiac surgery for patients with IHD is unclear. Mcfalls et al. reported that coronary artery revascularization before elective major vascular surgery did not improve long-term survival or reduce incidences of early postoperative outcomes—including death, myocardial infarction, and duration of hospital stay—among patients with stable coronary disease [24]. Another study found that preoperative coronary revascularization was not associated with improved outcomes, even for high-risk patients with disease affecting two or three vessels or left main disease [25]. In contrast, the guidelines of the Japanese Circulation Society recommend coronary revascularization before non-cardiac surgery for patients with unstable angina and those with stable angina with left main coronary artery disease, severe triple-vessel disease, or double-vessel disease affecting the proximal left anterior descending artery and low left ventricular ejection fraction [23]. The need for coronary revascularization should be individually assessed. To determine the most appropriate treatment strategy, an anesthesiologist and cardiologist should be consulted.

**Table 2:** Revised Cardiac Risk Index

Ischemic heart disease (history of myocardial infarction, positive exercise test, current complaint of ischemic chest pain or use of nitrate therapy, or ECG with Q waves)		
History of heart failure		
History of cerebrovascular disease (transient ischemic attack or cerebral infarction)		
Insulin therapy for diabetes		
Renal dysfunction (serum creatinine > 2.0 mg/dL)		
High-risk surgery (major vascular surgery)		

Number of risk factors	Cardiovascular complications (%) (95% CI)	Cardiovascular death (%)
0	0.5 (0.2–1.1)	0.3
1	1.3 (0.7–2.1)	0.7
2	3.6 (2.1–5.6)	1.7
≥ 3	9.1 (5.5–13.8)	3.6

*CI* confidence interval

##### Strategy for patients requiring percutaneous coronary intervention (PCI) before non-cardiac surgery

Elective surgery that has a high risk of intra- or postoperative surgery bleeding should be avoided for 12 months after implantation of drug-eluting stents (DES) and for at least 1 month after implantation of bare-metal stents (BMS). However, for malignant disease such delays should be minimized to prevent disease progression. Therefore, indications for DES implantation should be carefully considered in patients who have or are suspected to have malignant disease. BMS and balloon angioplasty are preferable to DES implantation for patients who require surgery within 12 months of PCI [23].

##### Perioperative management of patients who underwent PCI

A Japanese follow-up survey of 2,398 of 12,207 patients who had undergone PCI and underwent surgical treatment within 3 years found that 110 patients underwent respiratory surgery [26]. Stent thrombosis is a major concern, but the rate of restenosis is significantly lower for DES than for BMS. Dual antiplatelet therapy with aspirin and thienopyridine is the most beneficial regimen to prevent stent thrombosis and should continue for 12 months after DES implantation. Continuation of aspirin therapy during the perioperative period of non-cardiac surgery is recommended for patients already receiving aspirin [23].

Empirical perioperative use of heparin in patients with coronary stents is standard practice in many institutions in Japan, although there is no evidence that heparin prevents stent thrombosis. Guidelines vary regarding bridging with an anticoagulant agent. The Japanese Circulation Society maintains that heparinization is preferable when all antiplatelet agents must be discontinued [23]. In contrast, the American College of Chest Physicians does not recommend routine heparinization for patients who are receiving dual antiplatelet therapy and require surgery. Heparinization can be an obstacle in pain control by epidural anesthesia and may cause heparin-induced thrombocytopenia. Thus, perioperative antithrombotic therapy remains controversial, and future studies should assess the advantages and disadvantages of perioperative heparinization.

The frequency of stent thrombosis is lower for Japanese than for American and European populations. A Japanese study reported a cumulative incidence of definitive stent thrombosis of 0.6% at 1 year after sirolimus-eluting stent implantation [27]. The increase in the incidence of stent thrombosis during the 5 years after implantation was 0.2–0.3% per year. In European populations, the cumulative incidence of stent thrombosis for sirolimus-eluting stents and paclitaxel-eluting stents was 1.7% at 1 year and incidence continued to increase by 0.6% per year [28]. A recent report from Japan found that the incidence of stent thrombosis after lung resection was only 0.2% among patients with coronary stents, even though antiplatelet therapy had been discontinued in most patients [29]. Perioperative management of patients with coronary stents may differ for Japanese and American/European populations.

##### Concurrent coronary artery bypass surgery and lung cancer surgery

The effectiveness and feasibility of concurrent coronary artery surgery and pulmonary resection has been investigated [30]. The disadvantages of cardiopulmonary bypass can be avoided by using off-pump coronary artery bypass (OPCAB) grafting, a standard procedure for concomitant lung resection. Although clear indications have not been established for this concurrent operation, it might be optimal for patients with refractory stenosis of coronary arteries who cannot be treated with PCI. Another concern is the duration of antiplatelet therapy required between PCI and lung resection, as a delay in pulmonary surgery could allow cancer progression. Concurrent coronary artery bypass and lung cancer resection is an alternative therapeutic strategy for patients with advanced lung cancer requiring immediate resection.

#### Perioperative medical treatment for patients with cardiovascular disease

##### Statins

In addition to decreasing cholesterol synthesis, statins have pleiotropic effects, including vasodilation, anticoagulation, platelet inhibition, atherosclerotic plaque stabilization, and anti-inflammatory function. It has been reported that statins reduce mortality and cardiovascular outcomes in patients undergoing non-cardiac surgery. Moreover, statins were associated with reduced risks of respiratory, renal, and infection-related complications. A recent large-scale prospective cohort study indicated that preoperative statin therapy improved cardiovascular outcomes among patients undergoing non-cardiac surgery [31]. A retrospective observational cohort analysis of 180,478 veterans showed that early perioperative statin use significant reduced 30-day all-cause mortality and cardiovascular and non-cardiovascular complications, as compared with non-use [32]. These studies support the strong recommendation to continue perioperative statin use for non-cardiac surgery patients already receiving statins [22]. It remains to be determined whether statin treatment improves clinical outcomes for statin-naïve patients undergoing surgery. Although preoperative statin use might benefit statin-naïve patients undergoing cardiovascular surgery, there are insufficient data to support a definitive recommendation regarding perioperative statin therapy for patients undergoing non-cardiac surgery. The benefit of statins for statin-naïve patients is less clear, and ACC/AHA guideline includes less-than-strong recommendations to start perioperative statins for patients undergoing vascular surgery [22]. In contrast, the guidelines of the Japanese Circulation Society strongly recommend initiation of statin therapy for patients with high cardiovascular risk [23].

##### Beta-blockers

Beta-blockers can prevent myocardial infarction by prolonging coronary diastolic filling time, reducing myocardial oxygen consumption, and decreasing myocardial wall stress. Although some randomized controlled trials, a large-scale retrospective cohort study, and a meta-analysis showed a benefit for beta-blockers, several subsequent studies did not. The randomized trial over 8,000 patients undergoing non-cardiac surgery to extended-release metoprolol or placebo within 2–4 h before surgery; therapy was continued for 30 postoperative days [33]. Metoprolol reduced myocardial infarction risk, but increased risks of all-cause mortality and stroke. Thus, perioperative use of beta-blockers remains controversial. According to current ACC/AHA guideline, perioperative beta-blocker use is strongly recommended for patients receiving chronic treatment [22]. In the ACC/AHA guidelines, initiation of beta-blocker treatment before surgery receives a weak recommendation for patients with an intermediate or high risk of myocardial ischemia, as indicated by preoperative risk stratification. Current guidelines recommend against starting beta-blocker therapy on the day of surgery. Previous studies varied in the beta-blocker used, timing of initiation, and dose; thus, additional studies are necessary to determine the utility of perioperative beta-blockade.

##### Aspirin

Aspirin can prevent myocardial infarction in patients with coronary artery disease. Determining whether aspirin should be continued in patients undergoing non-cardiac surgery is a common clinical dilemma, as clinicians must balance the benefit of aspirin in preventing thromboembolic complications with the possibility of increased intra- and postoperative bleeding. The POISE-II trial randomly assigned over 10,000 patients undergoing non-cardiac surgery to receive aspirin or placebo [34]. Perioperative administration of low-dose aspirin did not reduce rates of mortality or myocardial infarction, although aspirin was associated with increases in major bleeding episodes. However, only 23% of patients in the POISE-II trial had coronary heart disease, and patients who received a BMS less than 6 weeks before surgery or a DES less than 1 year before surgery were excluded. Only 4.7% had a history of PCI and only 1.2% had a DES in this trial. Thus, this analysis cannot conclusively determine whether perioperative temporary aspirin cessation is optimal for patients in high-risk groups. A recent report from Japan suggested that discontinuation of antiplatelet therapy may not increase postoperative complications in patients with coronary artery disease who underwent pulmonary resection [29]. In that study of 902 patients who had received preoperative diagnoses of coronary artery stenosis, 532 patients (59%) had coronary stents and 204 patients (23%) were treated with DES. Nevertheless, stent thrombosis developed only in one patient in the study. Therefore, perioperative discontinuation of aspirin may be beneficial, even for patients with coronary stents. However, present Japanese guidelines recommend continuation of aspirin therapy during the perioperative period of non-cardiac surgery for patients already receiving aspirin and specify that perioperative aspirin use should be determined on an individual basis, after considering cardiovascular and perioperative hemorrhagic risks [23].

### ESRD

The number of lung cancer patients with ESRD is increasing, and perioperative risk is greater for such patients, regardless of hemodialysis (HD) status. ESRD patients not receiving HD lack dialytic support despite decreased renal excretion function. Monitoring of urinary output is thus necessary in order to determine the amount of fluid administration. Dosing of various medicines, including antibiotics, must be adjusted to renal function levels. Continuous hemodiafiltration should be considered when laboratory data suggest hyperkalemia or metabolic acidosis, or when pulmonary edema results from excessive volume [35]. Patients with severely impaired renal function should receive perioperative HD.

The number of ESRD patients on HD is increasing in many countries because the prognosis for these patients has improved as a result of medical progress and changes in trends regarding cause of death [36–38]. The incidence of cardiovascular disease, the main cause of death for hemodialysis patients, has decreased, but the incidences of other diseases, such as infection and malignant disease, have not [38]. The probability of treatment has increased during the past decade because of the better prognosis of ESRD patients.

#### Perioperative risk for hemodialysis patients

Perioperative mortality and morbidity are higher for ESRD patients receiving hemodialysis after surgery than for non-ESRD patients. One of the largest multicenter studies on this subject was conducted by Gajdos et al. They used the American College of Surgeons National Surgical Quality Improvement Program database to identify 1,506 dialysis patients who underwent major non-emergent general surgery [39]. Dialysis patients were more likely than non-dialysis patients to develop adverse events within 30 days postoperatively (morbidity 28.6 vs 10.7%, respectively; *P* < 0.001; mortality 12.7 vs 1.5%; *P* < 0.001). Several small-scale studies [40–50] evaluated surgical risk associated with hemodialysis in patients undergoing pulmonary resection (Table 3), and a 2017 study of the Japan NCD clearly showed high mortality and morbidity in this population [1]. The small-scale studies showed that hyperkalemia was the most frequent complication after pulmonary resection (Table 4).

**Table 3:** Case series of hemodialysis patients undergoing lung cancer surgery

Author	Year	No. of pts	Surgical procedure	Approach	Morbidity (%)
Ohta [40]	1992	1	Pn	Open	1 (100)
Yamamoto [41]	1996	1	Lb	Open	1 (100)
Tsuchida [42]	2001	7	Lb	Open	7 (100)
Ciriaco [43]	2005	6*	Lb 5, Pn 1, Weg 1	Open	4 (57)
Yajima [44]	2005	1	Lb	Open	1 (100)
Obuchi [45]	2009	11	Lb 9, Pn 1, Weg 1	VATS 4, Open 7	3 (27)
Akiba [46]	2010	2	Lb 1, Wed 1	VATS	0 (0)
Takahama [47]	2010	24	Lb 22, Sg 1, Weg 1	Open	13 (59)
Matsuoka [48]	2013	5	Lb 4, Sg 1	VATS	2 (40)
Caroli [49]	2015	1	Lb	VATS	0 (0)
Park [50]	2015	7	Lb 5, Weg 2	VATS 4, Open 3	3 (42)

*No* number, *pts* patients, *Pn* pneumonectomy, *Lb* lobectomy, *Sg* segmentectomy, *Weg* wedge resection

*One patient had two operations

**Table 4:** Morbidities after lung cancer surgery in hemodialysis patients [40–50]

Complications	No. (%)
Hyperkalemia	8 (19)
Prolonged air leakage	6 (14)
Sputum retention	5 (13)
Atrial fibrillation	5 (13)
Pneumonia	4 (9)
Chronic heart failure	3 (7)
Pleural effusion	2 (5)
Shunt thrombosis	2 (5)
Chylothorax	2 (5)
Others	5 (13)
Total	42 (100)

#### Perioperative management

##### Preoperative evaluation

Cardiovascular evaluation is important before surgery, as approximately 50% of dialysis patients undergoing surgery have cardiovascular disease [51]. The mortality rate for dialysis patients with diabetes is 44 times that of the non-ESRD population; thus, preoperative cardiovascular evaluation is essential [52]. When cardiovascular disease is suspected, further assessment is necessary, including cardiac catheter testing or myocardial perfusion scintigraphy. Pulmonary hypertension is a risk factor for pulmonary resection and an independent predictor of mortality in hemodialysis patients [53]. Screening of pulmonary hypertension by echocardiography is essential before lung cancer surgery.

##### Surgical procedure

Minimally invasive surgery might decrease postoperative complications for hemodialysis patients, because greater blood loss and longer surgical time worsen postoperative outcomes [54]. Although there is no evidence regarding surgical procedure, we avoid pneumonectomy when possible, to limit the possibility of hypostasis. The range of lymph node dissection is considered for each patient. Lymph node sampling may be appropriate for early cancer, but systematic lymph node dissection may be an option for advanced cancer, because sequential chemotherapy is limited for dialysis patients.

##### Hemodialysis program

To prevent excessive acidosis, we routinely perform hemodialysis on the day before surgery at our institution. Chronic acidosis is common in ESRD patients, as the kidneys are unable to excrete the nonvolatile acid load [55]. However, major surgery often causes postoperative metabolic acidosis [56]. Particularly after pulmonary resection, respiratory compensation decreases and leads to severe postoperative metabolic acidosis, which is associated with the worst postoperative outcomes [57]. Therefore, strict acid–base control is crucial in ESRD patients.

##### Postoperative management

Hemodialysis should be resumed within 24 h after surgery, to avoid the risk of bleeding and insufficient fluid shifts, unless there is severe progression of acidosis or volume overloads requiring emergent dialysis. Heparin-free hemodialysis is performed to avoid postoperative hemorrhage.

Routine evaluation of arterial blood gasses is necessary for ESRD patients to prevent unexpected acidosis and hyperkalemia. Fluid management is also important. However, perioperative use of a pulmonary artery catheter for fluid management in non-cardiac surgery is not recommended [58]. Ultrasonographic evaluation of the diameter of the inferior vena cava may be a convenient technique to estimate intravascular volume [59].

Acetaminophen and opioids are the usual drugs for postoperative pain control. Fentanyl is the best choice for dialysis patients, because of its short redistribution phase [60]. In contrast, meperidine, morphine, and propoxyphene should be avoided. Use of nonsteroidal anti-inflammatory drugs (NSAIDs) should be considered if the patient has residual renal function.

The rate of surgical site infection associated with thoracic surgery was reported to be 0.7–2.0% [61], and the rate for ESRD patients is believed to be much higher. Antibiotic prevention of surgical site infection is administered within 1 h before the initial incision, and cefazolin is recommended for hemodialysis patients undergoing thoracic surgery. There is no consensus regarding postoperative surgical antibiotic prophylaxis prolongation for ESRD or non-ESRD patients [5, 62]. Because antibiotic dose is limited for hemodialysis patients, we administered only once before postoperative hemodialysis.

##### Surgical outcome

Long-term outcomes are not as good for hemodialysis patients undergoing lung cancer surgery as for patients not receiving hemodialysis. Some small studies reported a 5-year overall survival rate of 28–43% [45, 47, 50], while 5-year overall survival after induction of hemodialysis was approximately 41.5–60.8% [36, 37]. Survival is worse for patients with diabetes than for those without diabetes (20.7–34%) [63]. It is unclear whether surgical intervention is indicated for diabetic ESRD patients with advanced lung cancer. Thus, further study is necessary.

In conclusion, we reviewed and discussed recent trends in specialized perioperative care for comorbidities such as IPF, CVD, and ESRD in patients undergoing lung cancer surgery. To improve surgical outcomes for lung cancer patients, future studies should attempt to identify optimal perioperative management strategies for these comorbidities.
